# Transactions of the State Medical Society of Wisconsin

**Published:** 1887-05

**Authors:** 


TRANSACTIONS OF THE STATE MEDICAL SOCIETY OF WISCONSIN, *1886, pp. 254.*

This volume is made up mostly of papers read at the meeting, some of which are of general interest. This society seems to prefer papers to reports of committees, and the preference seems to have some advantages. The volume contains the usual lists of officers of the society and their addresses, etc.

